# Predictive and Prognostic Role of Lipocalin-2 Expression in Prostate Cancer and Its Association with Gleason Score

**DOI:** 10.1155/2021/8836043

**Published:** 2021-01-20

**Authors:** M. Hakan Ulusoy, Yalcin Cirak, Yasemen Adali

**Affiliations:** ^1^Canakkale Onsekiz Mart University, School of Medicine, Department of Internal Medicine, Çanakkale, Turkey; ^2^Canakkale Onsekiz Mart University, School of Medicine, Department of Internal Medicine, Division of Medical Oncology, Çanakkale, Turkey; ^3^Izmir University of Economics, Faculty of Medicine, Department of Pathology, İzmir, Turkey

## Abstract

Lipocalin-2 has an important role in tumor progression, invasion, and metastasis. However, its role in prostate cancer remains unclear. The objective of this study is to determine the expression level of lipocalin-2 in human prostate cancer tissues and to evaluate the relationship between its expression level and clinicopathologic parameters including response to docetaxel treatment, Gleason score, progression-free survival (PFS), and overall survival (OS). We retrospectively analyzed paraffin-embedded tissue sections from 33 metastatic castrate-resistant prostate cancer (mCRPC) patients whose clinical outcomes had been tracked after docetaxel treatment. The expression status of lipocalin-2 was defined by immunohistochemistry (IHC) using the anti-lipocalin-2 antibody. Lipocalin-2 was highly expressed in 36% of the examined specimens. There was no significant correlation between high lipocalin-2 expression and docetaxel response (*p* : 0.09). High lipocalin-2 expression was signiﬁcantly associated with a higher Gleason score (*p*=0.027). Kaplan–Meier survival analysis failed to show a significant correlation between expression levels of lipocalin-2 and both OS and PFS although patients with high lipocalin-2 levels had a numerically shorter PFS and OS time compared to patients with low levels. Consequently, it is clear that further studies are needed to evaluate the predictive and prognostic role of lipocalin-2 in prostate cancer patients.

## 1. Introduction

Prostate cancer is the leading reason of death from cancer in men [1]. Despite the advances in treatment, many patients develop recurrence after radical prostatectomy or definitive radiotherapy. All recurrent and metastatic patients who initially respond to antiandrogen therapy inevitably progress to the castration-resistant status. In this clinical situation, docetaxel and new hormonal treatments (abiraterone and enzalutamide) are the main treatment options [2]. However, these treatment options are not effective for all metastatic castrate-resistant prostate cancers (mCRPC). About 50–60% of these patients do not benefit from docetaxel [3]. Therefore, it is necessary to develop a marker predicting sensitivity to docetaxel in order to deﬁne the patient group who will gain beneﬁt with docetaxel.

Lipocalin-2 (also known as oncogene 24p3, neutrophil gelatinase-associated lipocalin, and uterocalin) is a glycosylated protein originally purified from neutrophil granules [4, 5]. Lipocalin-2 functions as a transporter of some lipophilic molecules, such as retinoids, fatty acids, and cholesterol. Moreover, it is involved in cell differentiation, iron delivery, cell migration, and apoptosis. The best-characterized function of lipocalin-2 is to deprive bacteria of iron essential to their growth. Thus, lipocalin-2 plays an important role in the innate immune response against bacterial infection [6, 7]. Surprisingly, lipocalin-2 expression has been shown to be increased in many types of cancer in recent years, including esophageal [8, 9], pancreas [10], ovarian [11], breast [12], lung [13], and gastric [14] cancer. It increases the activity of matrix metalloproteinase 9 (MMP9), which breaks down the extracellular matrix and basement membranes, by preventing its autodegradation, and results in tumor progression, invasion, and metastasis [15].

Previous in vitro studies have shown that lipocalin-2 overexpression facilitates the progress of CRPC by increasing androgen receptor transcriptional activity [16]. In addition, in the xenograft prostate cancer model, lipocalin-2 overexpression has significantly promoted tumor growth. In another study, it has been reported that high lipocalin-2 expression correlated significantly with tumor differentiation (*p* < 0.017) and Gleason's grade [17]. In addition, previous studies have reported that expression level of lipocalin-2 in cancer patients may be associated with survival. In our study, we retrospectively evaluated the lipocalin-2 expression immunohistochemically in sections obtained from parafﬁn-embedded human prostate adenocarcinoma tissue samples, investigated the role of lipocalin-2 expression in predicting treatment response to docetaxel, and evaluated whether there is a relationship between its expression levels and Gleason's grade in the patients with metastatic prostate adenocarcinoma. Survival was also analyzed to determine the prognostic value of lipocalin-2 in these patients.

## 2. Methods

### 2.1. Patient and Tissue Samples

Cases were selected retrospectively from records of Canakkale Onsekiz Mart University Health Research and Practice Hospital and Canakkale Government Hospital between the years 2015–2019. The study has been approved by the Çanakkale Onsekiz Mart University Ethics Committee (approval number: 2019-17). Patients who were diagnosed for adenocarcinoma of prostate and administered at least three cycles of docetaxel (75 mg of docetaxel per square meter as a 1 h intravenous infusion on day 1 every 21 days together with 10 mg/day prednisone continue) for mCRPC to exclude prostate specific antigen (PSA) ﬂare as a bias source in the selection of patients were included into the study. All patients had regular measurements of PSA (every 3 weeks during active treatment). A total of 33 patients who met eligibility criteria were stratiﬁed according to treatment responses to docetaxel into two groups as responders and nonresponders. In the responder group (patients with response, partial response, stable disease, and ﬂare phenomena) PSA response was deﬁned according to the prostate-speciﬁc antigen working group consensus criteria [18]:

- Response: PSA decline ≥50% from baseline.
- Partial response and stable disease: PSA decline <50% but >0.
- Flare phenomena: initial PSA elevation followed by a decline below the PSA baseline (including patients with PSA decline ≥50% from baseline).

Disease progression under docetaxel treatment (nonresponder group) was deﬁned according to the Prostate Cancer Working Group (PCWG3 2015) [19] criteria as continuous, irreversible rise of PSA (two consecutive PSA increase of 25% and 2 *µ*g/l above the nadir) despite therapy and/or progression of measurable disease according to the Response Evaluation Criteria in Solid Tumor (RECIST) and/or appearance of 2 or more new bone lesion. In addition, patients were stratiﬁed into two groups according to Gleason score as grade group 1–4 (Gleason score ≤8) and grade group 5 (Gleason score 9-10).

### 2.2. Immunohistochemical Evaluation

**Figure 1:**
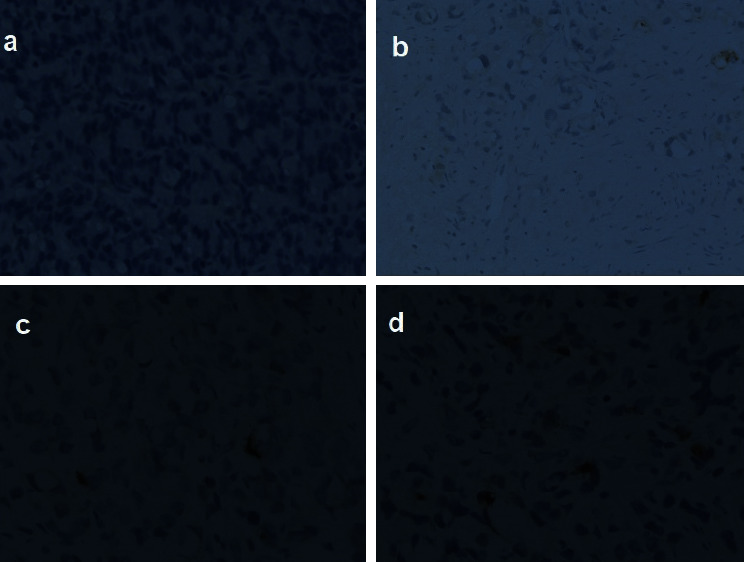
Immunohistochemistry staining using the primary antibody against lipocalin-2: (a) negative, 400x; (b) weak, 200x; (c) moderate, 400x; (d) strong expression, 400x.

**Figure 2:**
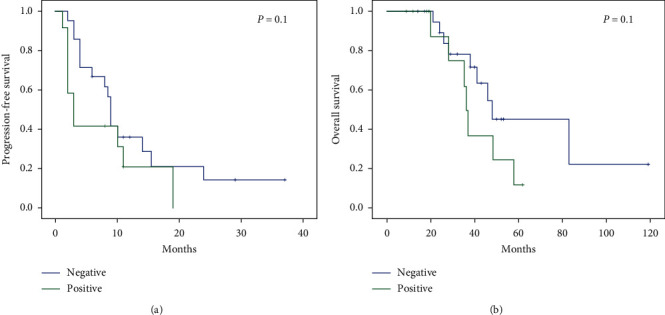
Progression-free survival (a) and overall survival (b) in lipocalin-2-positive and lipocalin-2-negative patients.

Formalin-fixed and paraffin-embedded tissue specimens of all patients were obtained from pathology department archive of Canakkale Onsekiz Mart University Health Research and Practice Hospital and Canakkale Government Hospital, and these paraffinized tissues were used for IHC staining. These specimens were cut (4 *μ*m) and stained with hematoxylin and eosin. A representative slide of each case was stained with lipocalin-2 antibody (Sigma Aldrich clon PA348-26.3.5,1/1000 dilution) in the Leica Bond Max fully automated IHC device (Leica Biosystems). IHC staining was carried out according to the manufacturer's instructions for both the antibody and device. Stomach tissue was used as a positive control, and a section without primary antibody was used as a negative control. Lipocalin-2 expression was generally cytoplasmic. As normal prostate epithelium showed weak staining with lipocalin-2 antibody, lipocalin-2 expression on tumor cells was evaluated according to a score corresponding to the sum of both: (a) staining intensity (0, negative (Figure 1(a)); 1, weak (Figure 1(b)); 2, intermediate (Figure 1(c)); and 3, strong (Figure 1(d))) and (b) percentage of positive cells (0, 0% positive cells; 1, <25% positive cells; 2, 26–50% positive cells; and 3, >50% positive cells), as described elsewhere [20]. The sum of a + b reached a maximum score of 6. Slides were scored in the absence of any clinical data, and a score 3 and greater than 3 was evaluated as high expression.

### 2.3. Statistics

Each clinicopathological variable was compared between the lipocalin-2-positive and -negative expression groups and evaluated with *X*^2^ test or Fisher's exact test. OS time was calculated using the Kaplan–Meier method as the time from the date of diagnosis to the date of death or last follow-up. Differences between low and high expression groups were compared using the log-rank test. *p* < 0.05 (two-tailed) was considered statistically significant. All statistical analyses were performed using SPSS, version 20.

## 3. Results

### 3.1. Lipocalin-2 Expression in Prostate Cancer and Its Relationship to the Clinical Effectiveness of Docetaxel

Twelve tumors (36%) showed positive expression for lipocalin-2. Of all patients, 60% (20/33) were responsive to docetaxel. Of patients with lipocalin-2-positive tumors, 41% (5/12) had docetaxel-responsive disease, compared with 71% (15/25) of lipocalin-2-negative tumors (*p*=0.095). There was no significant association between the lipocalin-2-positive and lipocalin-2-negative groups with respect to the clinical effectiveness of docetaxel. The association between the expressions level of lipocalin-2 and clinicopathological parameters is summarized in Table 1.

### 3.2. Association of Lipocalin-2 Expression with Gleason Score

Of lipocalin-2-positive tumors, 83% (10/12) had a Gleason score of 9 or 10 (grade group 5), compared with 42% (9/21) of lipocalin-2-negative tumors (*p*=0.027). There was significant association between the expression levels of lipocalin-2 protein and Gleason score (Table 1).

### 3.3. Association of Lipocalin-2 Expression with Survival

PFS was defined as the time from treatment initiation with docetaxel to disease progression or last visit. All but seven patients in the study group showed progression at the time of analysis. The longest PFS was 37 months. Median PFS was 13, 1 month in patients with lipocalin-2-negative tumors compared to 7, and 4 months for those with lipocalin-2-positive tumors (*p*=0,14). There was no signiﬁcant difference between two groups with respect to PFS. Sixteen of 33 patients (48%) died from the date of diagnosis to the last follow-up. The longest OS was 119 months. The median survival time in patients with positive and negative lipocalin-2 expression was 40.5 and 65.7 months, respectively (log-rank test, *p*=0.16, Figure 2).

## 4. Discussion

Docetaxel which is a microtubule-stabilizing agent leads to defective spindle formation by increasing polymerization and inhibiting depolymerization of microtubules. Defective spindle formation activating the mitotic checkpoint results in cell cycle arrest and apoptosis [21]. Various molecules such as microtubule-associated proteins, tau, Aurora A, spindle assembly checkpoint proteins (MAD1-3, BUB1-3, and BubR1) and class III *β*-tubulin have been reported as predictive markers for microtubulising agents [22].

A previous in vitro study showed that lipocalin-2 gene was one of the highly amplified genes in paclitaxel-resistant PC-3-TxR cell obtained from parent prostate cancer cell line (PC-3) [23]. However, there is only one clinical study investigating the predictive role of lipocalin-2 in the current literature. In this study, it has been reported that lipocalin-2 was a predictive marker for pathological complete response after neoadjuvant chemotherapy [24] in the low-risk subgroup of breast cancer. In this clinicopathological study that we conducted to confirm the in vitro result and determine the prognostic role of lipocalin-2 in prostate cancer patients, statistical analysis failed to show significant association between the levels of lipocalin-2 expression and the effectiveness of docetaxel although patients with high lipocalin-2 expression were more resistant to docetaxel (*p*=0.09).

In some studies, contradictory results have been reported for the association between some clinicopathologic parameters such as tumor differentiation, tumor stage, and lymph node metastasis and lipocalin-2 expression. Pancreatic cancer study [10] reported that high expression of lipocalin-2 was related to negative lymph node metastasis and earlier TNM stage. Conversely, cervical [25], colorectal [26], ovarian [27], and breast cancer studies reported that the high level of lipocalin-2 expression was associated with poor differentiated tumors, higher lymph node metastasis, and higher tumor stage. In addition, two in vitro studies conducted in oral squamous cell carcinoma (OSCC) and colon carcinoma cell line have been shown that lipocalin-2 expression reduces metastatic potential of these cells [28, 29]. There are only two studies investigating the relationship between prostate cancer and lipocalin-2 expression in the current literature. Tung MC et al. showed that high lipocalin-2 expression was significantly correlated with tumor differentiation (*p* < 0.017) and Gleason's grade, and knockdown of lipocalin-2 suppresses the growth and invasion of prostate cancer cells [17]. In their study, lipocalin-2 was highly expressed in 37% of tissues of prostate cancer according to normal prostate tissues. Similarly, in our study, we found that lipocalin-2 was highly expressed in 36% of patients, and high lipocalin-2 expression was significantly associated with higher Gleason score (*p*=0.027).

Different results have also been reported for the relationship between lipocalin-2 expression and survival. OSCC [28] and pancreatic cancer [10] study reported that high lipocalin-2 expression was related to a longer survival time. Conversely, cervical, lung, colon, ovarian, and breast cancer studies reported that high lipocalin-2 expression significantly correlated with a shorter survival time [13, 25–27, 30]. There is no clinical study investigating the relationship between the lipocalin-2 expression in patients with prostate cancer and survival in the current literature, and unfortunately, we failed to show a correlation between the lipocalin-2 expression and both PFS and OS in our study. However, patients with high lipocalin-2 expression had a numerically shorter PFS and OS time compared to patients with low levels.

To the best our knowledge, this is the first study investigating the effect of lipocalin-2 expression upon survival and clinical effectiveness of docetaxel treatment. However, some limitations such as the presence of censored data, the small size of the sample, and difficulties in the interpretation of IHC evaluation can be listed among the weaknesses of the study.

## 5. Conclusion

In summary, lipocalin-2 was highly expressed in about 36% of patients with prostate adenocarcinoma. High lipocalin-2 expression was signiﬁcantly associated with a higher Gleason score. Although no statistical significance was observed, patients with high lipocalin-2 expression tended to have lower effectiveness of docetaxel and shorter survival time. Despite significant correlation with Gleason score, our results did not support the predictive and prognostic role of lipocalin-2. It is clear that further studies are needed to evaluate the predictive and prognostic role of lipocalin-2 in prostate cancer patients.

## Figures and Tables

**Table 1:** Associations between the clinicopathological variables and lipocalin-2 expression.

Variable	*n*	Lipocalin-2 expression		*p* value
		Low	High	
Responders	20	15	5	0.09
Nonresponders	13	6	7	

Grade group 1–4	14	12	2	0.02
Grade group 5	19	9	10	

## Data Availability

The clinocopathologic data used to support the findings of this study are restricted by the Ethics Board of the Canakkale Onsekiz Mart University, School of Medicine, in order to protect patient privacy. Data are available from corresponding author for researchers who meet the criteria for access to confidential data.
